# Watching eyes take shape

**DOI:** 10.1016/j.gde.2015.02.004

**Published:** 2015-06

**Authors:** Naiara Bazin-Lopez, Leonardo E Valdivia, Stephen W Wilson, Gaia Gestri

**Affiliations:** Department of Cell and Developmental Biology, UCL, Gower Street, London WC1E 6BT, United Kingdom

## Abstract

Vertebrate eye formation is a multistep process requiring coordinated inductive interactions between neural and non-neural ectoderm and underlying mesendoderm. The induction and shaping of the eyes involves an elaborate cellular choreography characterized by precise changes in cell shape coupled with complex cellular and epithelial movements. Consequently, the forming eye is an excellent model to study the cellular mechanisms underlying complex tissue morphogenesis. Using examples largely drawn from recent studies of optic vesicle formation in zebrafish and in cultured embryonic stem cells, in this short review, we highlight some recent advances in our understanding of the events that shape the vertebrate eye.

**Current Opinion in Genetics & Development** 2015, **32**:73–79

This review comes from a themed issue on **Developmental mechanisms, patterning and organogenesis**

Edited by **Deborah J Andrew** and **Deborah Yelon**

For a complete overview see the Issue and the Editorial

Available online 3rd March 2015

**http://dx.doi.org/10.1016/j.gde.2015.02.004**

0959-437X/© 2015 The Authors. Published by Elsevier Ltd. This is an open access article under the CC BY license (http://creativecommons.org/licenses/by/4.0/).

## Introduction

Cells destined to form the eye occupy a single neuroectodermal domain, the eye field, which is specified within the anterior neural plate (ANP) through the action of a variety of signalling pathways that regionalize the forming CNS along its anterior to posterior and dorsal to ventral axes [1, 2, 3]. Eye field specification is determined by a conserved set of eye field transcription factors (EFTFs) [4] that promote eye identity, at least in part, by local repression of inductive signals that promote alternative ANP fates [2], and by regulating the changes in cell polarity, shape and movements that accompany eye formation [5^••^]. Indeed, the remarkable capacity of Pax6, one of the EFTFs, to promote eye formation across the animal kingdom helped to define the idea of the ‘Master Regulator’ [6]. While it is now clear that it is rarely true that a single gene can impart all aspects of cellular identity, it does seem likely that a relatively small number of EFTFs are sufficient to trigger neuroepithelial cells to form eyes.

Subsequent to eye field specification, shaping of the eye begins with the evagination of the optic vesicles. Upon contact with the overlying surface ectoderm the optic vesicle invaginates to form a double-layered optic cup with the internal neural retina and external retinal pigment epithelium (RPE) [7]. Further invagination of the optic cup forms a transient ventral opening, the choroid fissure [8]. Remarkably, many aspects of eye morphogenesis can be recapitulated *in vitro*, where three-dimensional embryonic stem (ES) cell cultures can be coaxed to self-organize into optic cups [9••, 10]. This suggests that EFTF-specified properties intrinsic to the eye tissue are sufficient to drive the epithelial movement and folding events that shape the optic cup. However, the bilateral evagination that splits the eye field *in vivo*, as well as formation and subsequent fusion of the choroid fissure are dependent on tissue-tissue interactions [7, 11, 12, 13], indicating that the environment does modulate the morphogenetic programme that generates functional eyes.

## The eye field specification programme initiates eye morphogenesis and segregates eye fated cells from adjacent neural plate territories

**Figure 1:**
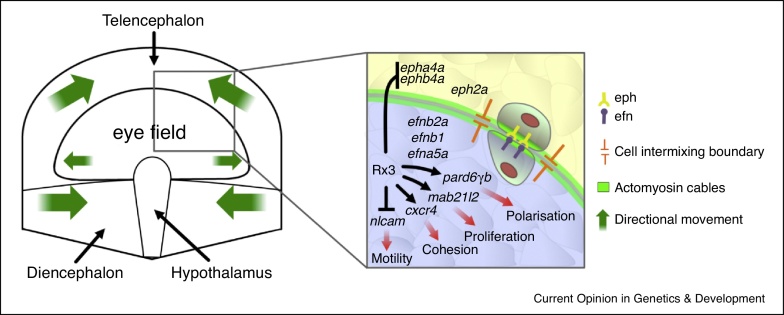
Eye field cells have different behaviours to, and do not intermix with, cells in adjacent neural plate domains. Left: Schematic representation of prospective forebrain territories at neural plate stage highlighting the eye field-telencephalon boundary. Eye field cells start to evaginate laterally (small green arrows) at the same time that most anterior neural plate cells are still converging towards the midline (large green arrows). The inset highlights the eye field-telencephalon boundary: Rx3 regulates genes that influence cell behaviours in the eye field. For instance, it restricts the expression of at least two *eph* genes to neural plate territories surrounding the eye and Eph/ephrin signalling subsequently maintains segregation between eye field cells and adjacent neural plate territories. Rx3 also controls the expression of genes that mediate discrete cell behaviours in the eye field.

The eye field undergoes a programme of morphogenesis that is distinct from adjacent neural plate domains. Consequently, establishing robust boundaries between the eyefield and adjacent forebrain domains is likely to be important to maintain sharp boundaries of EFTF gene expression and prevent eye field cells from mixing with surrounding cells, despite the extensive cell reorganization within the ANP (Figure 1) [5••, 14•, 16]. Thus, one role for the EFTFs may be to regulate expression of genes that ensure the eye field remains discrete from adjacent territories. Indeed this seems to be the case for Rx3, a transcription factor essential for eye formation across vertebrates [17, 18, 19, 20].

Among the genes mis-regulated in absence of *rx3* function in zebrafish are members of the Eph and ephrin families, which control the segregation of eye field cells from other ANP domains [15•, 21]. *Ephs* and *ephrins* encode transmembrane proteins involved in adhesion and repulsion processes upon direct cell–cell contact during development [22]. Rx3 contributes to establishing the complementary expression patterns of *Eph* and *ephrin* within the ANP, and abrogation of Eph/ephrin signalling leads to eye cells inappropriately intermixing with other neural plate cells, without affecting eye field specification (Figure 1) [15^•^]. These observations suggest that Eph/Ephrin pathway activation takes place at the border between the eye field and adjacent ANP domains. At inter-rhombomeric boundaries, Eph/ephrin signalling regulates the actomyosin cytoskeleton to establish mechanical barriers [23], and as accumulation of actomyosin cables is observed at the margins of the eye field [15^•^], this mechanism may also contribute to segregation of the eye field from adjacent ANP domains.

Rx3 also regulates the region-specific morphogenetic programme that causes eye field cells to bulge out laterally instead of converging towards the midline as other ANP cells do. Live imaging studies have shown that *rx3*-expressing cells exhibit slower midline convergence compared to neighbouring telencephalic and diencephalic cells [18]. This eye field-specific motile behaviour is influenced by Nlcam, a member of the immunoglobulin-superfamily of cell adhesion molecules. Rx3 maintains low levels of *nlcam* expression in the eye field compared to the adjacent ANP domains and this appears to be necessary for normal evagination (Figure 1) [24]. How Nlcam modulates migratory behaviours of eye field and ANP cells is not known.

Gene expression profiling studies have identified additional genes regulated by *rx3* [25, 26], including *mab21l2* and *cxcr4a*, which influence proliferation and cohesion of eye field cells, respectively (Figure 1) [14•, 24, 27]. As when Eph/ephrin signalling is disrupted, *cxcr4a* mutants show intermixing of eyefield and telencephalic cells [14^•^] and it will be of interest to resolve if these two pathways interact. Given the relatively small number of known Rx3 targets, more work is needed to identify other EFTF effectors regulating eye field specification, segregation and morphogenesis. Indeed, recent work in zebrafish has linked the Semaphorin/Plexin pathway to eye morphogenesis [28]. This signalling pathway, well characterized in cell migration and axon guidance, regulates a tissue-autonomous mechanism for cell cohesion within the optic vesicle. As for the Eph/ephrin pathway, disruption of Semaphorin/Plexin signalling does not impair eye field specification, suggesting that this pathway is another morphogenetic effector of the eye field transcriptional network.

While live imaging has proven central to understanding the cell behaviours regulated by the EFTFs to drive early eye morphogenesis in fish [15•, 18], mammalian embryos are not easily amenable to such imaging techniques. Recently, ES cell aggregates forming eye organoids have emerged as an appealing system to dissect cellular events accompanying eye specification and morphogenesis [9••, 10]. Such cultures should be amenable to imaging and offer the potential for using ES cells carrying genetic lesions to elucidate the contribution of the EFTFs in regulating mammalian eye morphogenesis.

## Basal lamina-dependent coordination of epithelial apico-basal polarity contributes to eye morphogenesis

In zebrafish, the early steps in formation of the CNS, such as convergence of neural plate cells towards the midline and anterior–posterior extension, occur prior to full epithelialization of the neural plate [29]. Indeed, full acquisition of apico-basal polarity usually occurs only after cells converge and undergo a midline crossing division [30]. The contemporaneous processes of tissue morphogenesis and acquisition of epithelial character may be related to the rapid speed at which teleost embryos develop; it is assumed that in amniotes, the neural plate is fully epithelialized prior to undergoing the morphogenetic movements that form the neural tube [31].

**Figure 2:**
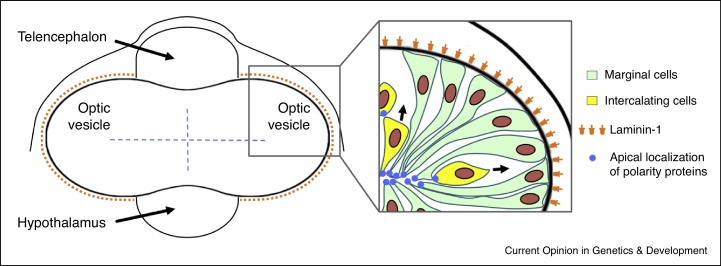
The optic vesicle is progressively epithelialized during morphogenesis in fish. Left schematic is a frontal view of the brain showing the evaginating optic vesicles. The blue dashes indicate the internal position of ventricular space within the optic vesicles and orange dots indicate abundant Laminin. The inset shows the organization of neuroepithelial cells in the evaginating optic vesicles. Cells at the margin of the eye field (pale green) show coordinated epithelial organization dependent upon contact with a Laminin-1 enriched basal lamina. Core cells (yellow) intercalate into the nascent epithelium aligning their apicobasal polarity and shape with their polarized neighbours during this process. Based on Ivanovitch *et al.* [5^••^].

Recent work has shown that the eye field in zebrafish displays precocious epithelialization compared to other domains of the neural plate [5^••^]. Consequently for some time, genetic programmes regulating apico-basal polarization and epithelial remodelling may be active in the eye field but not in adjacent neural plate domains (Figure 2). Indeed Laminin-1 is accumulated around the nascent optic vesicles before being detected elsewhere and there is spatially restricted expression in the eye field of other regulators of morphogenesis such as *pard6γb*, which encodes an apical polarity protein. Precocious *pard6γb* expression in the eye field is lost in *rx3* mutants [5^••^], suggesting that Rx3 activity may advance the developmental timer that initiates acquisition of apicobasal polarity in the neural plate [32].

Although all eye field cells express apicobasal polarity markers, it is only those cells at the leading surface of the outpocketing optic vesicles that coordinate their polarity and form a coherent neuroepithelial sheet [5^••^]. This suggests that epithelialization may be a prerequisite for the cell movements that accompany evagination. Coordination of apico-basal polarity between cells in the forming optic vesicle is dependent upon the underlying Laminin-1-rich basal lamina (Figure 2) [5^••^] as it is in other regions of the CNS [33]. Consequently, when Laminin-1 is absent, most neuroepithelial cells still polarize but fail to elongate and align with their neighbours with some showing completely reversed orientation of polarity [5^••^]. This appears to be a highly conserved role for the basal lamina in various other developmental contexts [34, 35]. For instance, ES cell organoids require Laminin rich extracellular matrix (Matrigel) to form epithelialized optic vesicles and other CNS structures [9••, 36, 37]. Similarly, the basal lamina coordinates polarization of epiblast cells as they form rosette-like structures during an early phase of mouse development [38].

One possible mechanism regulating the establishment of apicobasal polarity upon contact with the ECM was recently uncovered in cells forming epithelial hollow cysts when cultured in Laminin-rich matrigel [39^•^]. At the ECM-abutting plasma membrane, integrin–ECM interactions trigger local RhoA inactivation and protein kinase C (PKC)-dependent phosphorylation. This leads to transcytosis of Podocalyxin complexes to the apical membrane initiation site at the central core of the group of cells, thus initiating apical lumen formation [39^•^]. Consequently, the ECM could trigger a similar molecular mechanism for orienting polarity during development.

As cells at the margin of the eye field epithelialize, those located at its core remain mesenchymal in morphology [5^••^], presumably because they have yet not encountered a basal lamina. As evagination proceeds, these core cells undergo behaviours akin to a mesenchymal to epithelial transition in which they intercalate into the epithelialized marginal domain of the forming optic vesicle (Figure 2). The role of these cells in driving evagination is not known. Indeed, we have yet to make any significant insights into the driving forces that shape the forming optic vesicles. Although individual cell migration has been proposed to drive optic vesicle evagination in medaka [18], an alternative interpretation is that these migratory cells are equivalent to the core cells described in zebrafish that contribute to, but do not lead, evagination. Although tissue growth contributes to shape epithelia [40], blocking cell proliferation during eye development in Xenopus and zebrafish does not overtly affect morphogenesis [41, 42]. Consequently it is important to determine the biomechanical forces that contribute to optic vesicle formation to elucidate how this process is developmentally regulated, potentially using tools for visualizing and measuring such forces *in vivo* [43, 44, 45, 46].

**Figure 3:**
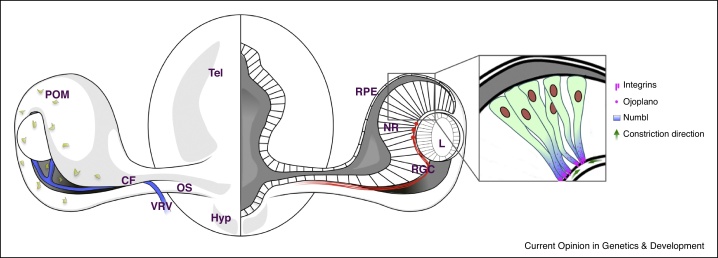
Generation of the choroid fissure during optic cup formation. Schematic frontal view of the forebrain showing the eyes and (inset) polarized retinal neuroepithelial cells during optic cup morphogenesis. The left side of the main figure shows a surface view of the optic vesicle and ventrally positioned choroid fissure. POM cells (green) surround the optic cup, invade the choroid fissure and later form the blood vessels of the eye (blue). The right side of the main figure shows a slice through the optic cup at the level of the choroid fissure. Retinal ganglion cell axons (red) use the fissure as a route out of the eye as they navigate towards central targets in the brain. The inset shows a higher resolution view of neuroepithelial cells in the neural retina. The basal end feet of these cells localize Integrin in an Ojoplano and Numb/Numbl dependent mechanism, and their constriction (green arrows) is thought to contribute to neuroepithelial bending and the invagination process. Based largely on Martinez-Morales *et al.* [48] and Bogdanović *et al.* [49^•^]. Abbreviations: CF, choroid fissure; Hyp, hypothalamus; L, Lens; NR, neural retina; OS, optic stalk; POM, periocular mesenchyme; RPE, retinal pigment epithelium; RGC, retinal ganglion cells; Tel, telencephalon; VRV ventral retinal blood vessels.

Modifying the relative size of the apical, lateral and basal domains of epithelial cells can lead to both evagination and invagination of epithelial tissues [47]; this process seems to be critical for the invagination accompanying the transition from optic vesicle to optic cup. *Ojoplano* mutant medaka fish exhibit severe invagination defects due to mis-regulation of Integrin trafficking that normally maintains a small basal domain in prospective neural retinal cells undergoing invagination (Figure 3) [48]. *Ojoplano* encodes a transmembrane protein that localizes basally in the retinal neuroepithelium, and antagonizes the Numb/Numbl pathway-mediated endocytosis of Integrin [49^•^]. As a consequence, *ojoplano* mutants display increased integrin-β1 internalization, which is proposed to affect transmission of cortical tension and cell shape changes across the retinal epithelium. Inwardly directed epithelial folding also requires Integrin-mediated activity during optic cup morphogenesis in self-organizing ES cell organoids [10].

One implication of the observation that core cells intercalate widely throughout the evaginating optic vesicle [5^••^] is that although eye field cells are committed to form eyes, there is unlikely to be any fate restriction with respect to which parts of the eye they form. At early stages of eye field formation, there is no evidence of spatially restricted expression of markers of prospective regional fates within the optic vesicle such as RPE and optic stalk. Indeed, there is a remarkable degree of movement of cells between different domains of the forming eyes. For instance cells positioned in the outer layer of the optic cup continue to migrate around the marginal rim of the cup into the neural retina until late stages of development [42, 50, 51•] and similarly, cells move from the optic stalk into ventral neural retina [52]. Coincident with these morphogenetic movements, cells are exposed to different environments and signals that influence their identity. For instance, cells that eventually form the nasal neural retina are influenced by Fgf signals coming from telencephalon, olfactory placode and parts of the optic vesicle itself at different stages during the morphogenesis process [51^•^]. Such observations suggest that morphogenesis and regional patterning are inherently linked to each other so that an eye field cell's eventual fate is the result of its trajectory and encounters during the process of optic cup morphogenesis. One caveat is that although fate determination is likely to occur concomitantly with the morphogenetic regionalization of the eye, there have not been experiments to directly test if there is any fate restriction among eye field cells in fish. Surprisingly, such restrictions may indeed be present in Xenopus as there do appear to be biases in the retinal neuron cell types derived from different blastomeres [53, 54, 55]. This suggests that eye field cells may not be a homogenous population in amphibia; whether there are biases in the ability of such cells to contribute to optic stalk, neural retina and RPE (as apposed to different neuron classes in the neural retina) has not been tested.

## Tissue interactions are critical to make functional eyes *in vivo*

In vertebrate embryos, coordinated interactions between tissues influence eye morphogenesis and patterning to ultimately generate a pair of functional eyes. Substantial aspects of this process cannot, as yet, be recapitulated *in vitro*, as environmental signals and constraints are absent. For instance, whereas optic vesicle evagination *in vivo* is bilateral and depends on signals from, and cell movements within, the axial mesoderm and neural ectoderm to split the eye field [56, 57, 69], each Rx positive domain in ES cell organoids generates a single optic cup [9^••^]. Although the mechanisms that split the eye field are not well understood, it is likely that mesodermal signals influence cell fate rather than (or perhaps in addition to) directly influencing movement of eye field cells. Indeed, axial signals promote proximal, optic stalk gene expression, while repressing the distal, prospective retinal gene expression [13, 58, 59]. Local modulation of axially derived signals, such as Shh and Nodal in ES cell optic vesicle organoids may provide a route to gain insights into the cellular mechanisms driving bilateral evagination, as well as proximo-distal patterning of the optic cup.

ES cells coaxed to form optic vesicles invaginate symmetrically to form a spherical optic cup whereas invagination *in vivo* is asymmetric, presumably due to environmental influences. Indeed, invagination progresses from the dorsal and most distal part of the optic vesicle, which contacts the lens forming ectoderm, ventrally and proximally along the forming retina and optic stalk [12]. This results in the formation of the choroid fissure, a transient opening along the ventral optic cup/stalk that allows entry of blood vessels and exit of retinal axons from the eye (Figure 3). The ventro-nasal and ventro-temporal lips of the choroid fissure subsequently fuse to close the globe of the eye and failure of these events cause coloboma [12]. The only example of symmetric invagination and absence of formation of a choroid fissure *in vivo* that we are aware of is in mice lacking BMP7 function [60]. It will be intriguing to resolve if optic cup formation in these mutants is similar to that seen in ES cell organoids that also lack choroid fissures.

Although a normal *in vivo* environment enables more complex eye morphogenesis than occurs in organoid cultures, abnormal *in vivo* environmental conditions can lead to formation of optic cups far more developmentally compromised than those formed from ES cell aggregates [9••, 10], or from optic vesicles transplanted to ectopic locations in the embryo [51^•^]. Consequently eye formation *in vivo* is both promoted and constrained by the environment in which optic cup morphogenesis occurs. For instance, Tfap2 is a transcription factor required for neural-crest dependent cranio-facial development [61, 62] and both fish and humans with compromised Tfap2 function show variable, severe abnormalities of eye formation [63]. It is presumed that this is due to disruption of the periocular mesenchyme (POM), which surrounds the forming optic cup (Figure 3). As optic cups can form *in vitro* in absence of POM, it seems likely that the major ocular phenotypes seen *in vivo* when Tfap2 is compromised are due to abnormal environmental architecture or signalling rather than an absolute requirement for POM in optic cup formation. Given the complexity of contemporaneous developmental events occurring in the vicinity of the forming eyes, there must be precise coordination of morphogenetic/migratory processes and signalling events and limited capability of the forming eye to cope with environmental disruption.

Although POM may not be essential for optic cup formation, it may well contribute to those aspects of morphogenesis such as choroid fissure formation and closure that, as yet, are not recapitulated in ES cell organoid cultures. POM invades the choroid fissure as it forms and closes and consequently POM cells are well positioned to contribute to this aspect of eye morphogenesis. Indeed, it appears that the fusion of the fissure is compromised when retinoid signalling is disrupted either in the lips of neural retina or in the surrounding POM [64, 65, 66]. What role the POM may play in this process is not yet known though one possibility would be in dissolution of the extracellular matrix that must occur before fusion of the two opposing neuroepithelial lips of the fissure.

One future avenue for investigation will be to ask if morphogenetic processes such as splitting of the eye field, choroid fissure formation and closure can be promoted in ES cell-derived optic cups through the provision of additional cell populations that influence eye formation *in vivo* (such as POM, lens and ventral CNS). In other contexts, organ cultures from ES cells can be facilitated by inclusion of additional cell types [67, 68]. If addition of other relevant cell types is able to influence eye formation from ES cells, then this may provide an excellent new model to study subtle aspects of normal eye morphogenesis and to elucidate why eye formation can be severely compromised in abnormal environments.

## References and recommended reading

Papers of particular interest, published within the period of review, have been highlighted as:

• of special interest
•• of outstanding interest

## References

[1] Beccari L., Marco-Ferreres R., Bovolenta P. (2013). The logic of gene regulatory networks in early vertebrate forebrain patterning. Mech Dev.

[2] Cavodeassi F., Houart C. (2012). Brain regionalization: of signaling centers and boundaries. Dev Neurobiol.

[3] Kiecker C., Lumsden A. (2012). The role of organizers in patterning the nervous system. Annu Rev Neurosci.

[4] Zuber M.E., Gestri G., Viczian A.S., Barsacchi G., Harris W.A. (2003). Specification of the vertebrate eye by a network of eye field transcription factors. Dev Camb Engl.

[5••] Ivanovitch K., Cavodeassi F., Wilson S.W. (2013). Precocious acquisition of neuroepithelial character in the eye field underlies the onset of eye morphogenesis. Dev Cell.

[6] Chow R.L., Altmann C.R., Lang R.A., Hemmati-Brivanlou A. (1999). Pax6 induces ectopic eyes in a vertebrate. Dev Camb Engl.

[7] Fuhrmann S. (2010). Eye morphogenesis and patterning of the optic vesicle. Curr Top Dev Biol.

[8] Chow R.L., Lang R.A. (2001). Early eye development in vertebrates. Annu Rev Cell Dev Biol.

[9••] Eiraku M., Takata N., Ishibashi H., Kawada M., Sakakura E., Okuda S., Sekiguchi K., Adachi T., Sasai Y. (2011). Self-organizing optic-cup morphogenesis in three-dimensional culture. Nature.

[10] Nakano T., Ando S., Takata N., Kawada M., Muguruma K., Sekiguchi K., Saito K., Yonemura S., Eiraku M., Sasai Y. (2012). Self-formation of optic cups and storable stratified neural retina from human ESCs. Cell Stem Cell.

[11] Adler R., Canto-Soler M.V. (2007). Molecular mechanisms of optic vesicle development: complexities, ambiguities and controversies. Dev Biol.

[12] Gestri G., Link B.A., Neuhauss S.C.F. (2012). The visual system of zebrafish and its use to model human ocular diseases. Dev Neurobiol.

[13] Lupo G., Harris W.A., Lewis K.E. (2006). Mechanisms of ventral patterning in the vertebrate nervous system. Nat Rev Neurosci.

[14•] Bielen H., Houart C. (2012). BMP signaling protects telencephalic fate by repressing eye identity and its Cxcr4-dependent morphogenesis. Dev Cell.

[15•] Cavodeassi F., Ivanovitch K., Wilson S.W. (2013). Eph/Ephrin signalling maintains eye field segregation from adjacent neural plate territories during forebrain morphogenesis. Dev Camb Engl.

[16] England S.J., Blanchard G.B., Mahadevan L., Adams R.J. (2006). A dynamic fate map of the forebrain shows how vertebrate eyes form and explains two causes of cyclopia. Dev Camb Engl.

[17] Loosli F., Staub W., Finger-Baier K.C., Ober E.A., Verkade H., Wittbrodt J., Baier H. (2003). Loss of eyes in zebrafish caused by mutation of chokh/rx3. EMBO Rep.

[18] Rembold M., Loosli F., Adams R.J., Wittbrodt J. (2006). Individual cell migration serves as the driving force for optic vesicle evagination. Science.

[19] Stigloher C., Ninkovic J., Laplante M., Geling A., Tannhäuser B., Topp S., Kikuta H., Becker T.S., Houart C., Bally-Cuif L. (2006). Segregation of telencephalic and eye-field identities inside the zebrafish forebrain territory is controlled by Rx3. Dev Camb Engl.

[20] Winkler S., Loosli F., Henrich T., Wakamatsu Y., Wittbrodt J. (2000). The conditional medaka mutation eyeless uncouples patterning and morphogenesis of the eye. Dev Camb Engl.

[21] Moore K.B., Mood K., Daar I.O., Moody S.A. (2004). Morphogenetic movements underlying eye field formation require interactions between the FGF and ephrinB1 signaling pathways. Dev Cell.

[22] Klein R. (2012). Eph/ephrin signalling during development. Dev Camb Engl.

[23] Calzolari S., Terriente J., Pujades C. (2014). Cell segregation in the vertebrate hindbrain relies on actomyosin cables located at the interhombomeric boundaries. EMBO J.

[24] Brown K.E., Keller P.J., Ramialison M., Rembold M., Stelzer E.H.K., Loosli F., Wittbrodt J. (2010). Nlcam modulates midline convergence during anterior neural plate morphogenesis. Dev Biol.

[25] Giudetti G., Giannaccini M., Biasci D., Mariotti S., Degl’innocenti A., Perrotta M., Barsacchi G., Andreazzoli M. (2014). Characterization of the Rx1-dependent transcriptome during early retinal development. Dev Dyn.

[26] Yin J., Morrissey M.E., Shine L., Kennedy C., Higgins D.G., Kennedy B.N. (2014). Genes and signaling networks regulated during zebrafish optic vesicle morphogenesis. BMC Genomics.

[27] Kennedy B.N., Stearns G.W., Smyth V.A., Ramamurthy V., van Eeden F., Ankoudinova I., Raible D., Hurley J.B., Brockerhoff S.E. (2004). Zebrafish rx3 and mab21l2 are required during eye morphogenesis. Dev Biol.

[28] Ebert A.M., Childs S.J., Hehr C.L., Cechmanek P.B., McFarlane S. (2014). Sema6a and Plxna2 mediate spatially regulated repulsion within the developing eye to promote eye vesicle cohesion. Dev Camb Engl.

[29] Clarke J. (2009). Role of polarized cell divisions in zebrafish neural tube formation. Curr Opin Neurobiol.

[30] Tawk M., Araya C., Lyons D.A., Reugels A.M., Girdler G.C., Bayley P.R., Hyde D.R., Tada M., Clarke J.D.W. (2007). A mirror-symmetric cell division that orchestrates neuroepithelial morphogenesis. Nature.

[31] Colas J.F., Schoenwolf G.C. (2001). Towards a cellular and molecular understanding of neurulation. Dev Dyn.

[32] Girdler G.C., Araya C., Ren X., Clarke J.D.W. (2013). Developmental time rather than local environment regulates the schedule of epithelial polarization in the zebrafish neural rod. Neural Dev.

[33] Buckley C.E., Ren X., Ward L.C., Girdler G.C., Araya C., Green M.J., Clark B.S., Link B.A., Clarke J.D.W. (2013). Mirror-symmetric microtubule assembly and cell interactions drive lumen formation in the zebrafish neural rod. EMBO J.

[34] Daley W.P., Yamada K.M. (2013). ECM-modulated cellular dynamics as a driving force for tissue morphogenesis. Curr Opin Genet Dev.

[35] Rodriguez-Fraticelli A.E., Martin-Belmonte F. (2014). Picking up the threads: extracellular matrix signals in epithelial morphogenesis. Curr Opin Cell Biol.

[36] Eiraku M., Watanabe K., Matsuo-Takasaki M., Kawada M., Yonemura S., Matsumura M., Wataya T., Nishiyama A., Muguruma K., Sasai Y. (2008). Self-organized formation of polarized cortical tissues from ESCs and its active manipulation by extrinsic signals. Cell Stem Cell.

[37] Suga H., Kadoshima T., Minaguchi M., Ohgushi M., Soen M., Nakano T., Takata N., Wataya T., Muguruma K., Miyoshi H. (2011). Self-formation of functional adenohypophysis in three-dimensional culture. Nature.

[38] Bedzhov I., Zernicka-Goetz M. (2014). Self-organizing properties of mouse pluripotent cells initiate morphogenesis upon implantation. Cell.

[39•] Bryant D.M., Roignot J., Datta A., Overeem A.W., Kim M., Yu W., Peng X., Eastburn D.J., Ewald A.J., Werb Z. (2014). A molecular switch for the orientation of epithelial cell polarization. Dev Cell.

[40] Keller R., Shook D. (2011). The bending of cell sheets – from folding to rolling. BMC Biol.

[41] Harris W.A., Hartenstein V. (1991). Neuronal determination without cell division in Xenopus embryos. Neuron.

[42] Kwan K.M., Otsuna H., Kidokoro H., Carney K.R., Saijoh Y., Chien C.-B. (2012). A complex choreography of cell movements shapes the vertebrate eye. Dev Camb Engl.

[43] Guo J., Sachs F., Meng F. (2014). Fluorescence-based force/tension sensors: a novel tool to visualize mechanical forces in structural proteins in live cells. Antioxid Redox Signal.

[44] Kashef J., Franz C.M. (2014). Quantitative methods for analyzing cell–cell adhesion in development. Dev Biol.

[45] Morimatsu M., Mekhdjian A.H., Adhikari A.S., Dunn A.R. (2013). Molecular tension sensors report forces generated by single integrin molecules in living cells. Nano Lett.

[46] Smutny M., Behrndt M., Campinho P., Ruprecht V., Heisenberg C.-P. (2015). UV laser ablation to measure cell and tissue-generated forces in the zebrafish embryo in vivo and ex vivo. Methods Mol Biol (Clifton, NJ).

[47] St Johnston D., Sanson B. (2011). Epithelial polarity and morphogenesis. Curr Opin Cell Biol.

[48] Martinez-Morales J.R., Rembold M., Greger K., Simpson J.C., Brown K.E., Quiring R., Pepperkok R., Martin-Bermudo M.D., Himmelbauer H., Wittbrodt J. (2009). Ojoplano-mediated basal constriction is essential for optic cup morphogenesis. Dev Camb Engl.

[49•] Bogdanović O., Delfino-Machín M., Nicolás-Pérez M., Gavilán M.P., Gago-Rodrigues I., Fernández-Miñán A., Lillo C., Ríos R.M., Wittbrodt J., Martínez-Morales J.R. (2012). Numb/Numbl-Opo antagonism controls retinal epithelium morphogenesis by regulating integrin endocytosis. Dev Cell.

[50] Li Z., Joseph N.M., Easter S.S. (2000). The morphogenesis of the zebrafish eye, including a fate map of the optic vesicle. Dev Dyn.

[51•] Picker A., Cavodeassi F., Machate A., Bernauer S., Hans S., Abe G., Kawakami K., Wilson S.W., Brand M. (2009). Dynamic coupling of pattern formation and morphogenesis in the developing vertebrate retina. PLoS Biol.

[52] Holt C. (1980). Cell movements in Xenopus eye development. Nature.

[53] Huang S., Moody S.A. (1995). Asymmetrical blastomere origin and spatial domains of dopamine and neuropeptide Y amacrine subtypes in Xenopus tadpole retina. J Comp Neurol.

[54] Huang S., Moody S.A. (1997). Three types of serotonin-containing amacrine cells in tadpole retina have distinct clonal origins. J Comp Neurol.

[55] Moody S.A., Chow I., Huang S. (2000). Intrinsic bias and lineage restriction in the phenotype determination of dopamine and neuropeptide Y amacrine cells. J Neurosci.

[56] Chiang C., Litingtung Y., Lee E., Young K.E., Corden J.L., Westphal H., Beachy P.A. (1996). Cyclopia and defective axial patterning in mice lacking Sonic hedgehog gene function. Nature.

[57] Hatta K., Kimmel C.B., Ho R.K., Walker C. (1991). The cyclops mutation blocks specification of the floor plate of the zebrafish central nervous system. Nature.

[58] Ekker S.C., Ungar A.R., Greenstein P., von Kessler D.P., Porter J.A., Moon R.T., Beachy P.A. (1995). Patterning activities of vertebrate hedgehog proteins in the developing eye and brain. Curr Biol CB.

[59] Macdonald R., Barth K.A., Xu Q., Holder N., Mikkola I., Wilson S.W. (1995). Midline signalling is required for Pax gene regulation and patterning of the eyes. Dev Camb Engl.

[60] Morcillo J., Martínez-Morales J.R., Trousse F., Fermin Y., Sowden J.C., Bovolenta P. (2006). Proper patterning of the optic fissure requires the sequential activity of BMP7 and SHH. Dev Camb Engl.

[61] Knight R.D., Nair S., Nelson S.S., Afshar A., Javidan Y., Geisler R., Rauch G.-J., Schilling T.F. (2003). Lockjaw encodes a zebrafish tfap2a required for early neural crest development. Dev Camb Engl.

[62] Schorle H., Meier P., Buchert M., Jaenisch R., Mitchell P.J. (1996). Transcription factor AP-2 essential for cranial closure and craniofacial development. Nature.

[63] Gestri G., Osborne R.J., Wyatt A.W., Gerrelli D., Gribble S., Stewart H., Fryer A., Bunyan D.J., Prescott K., Collin J.R.O. (2009). Reduced TFAP2A function causes variable optic fissure closure and retinal defects and sensitizes eye development to mutations in other morphogenetic regulators. Hum Genet.

[64] Lupo G., Gestri G., O’Brien M., Denton R.M., Chandraratna R.A.S., Ley S.V., Harris W.A., Wilson S.W. (2011). Retinoic acid receptor signaling regulates choroid fissure closure through independent mechanisms in the ventral optic cup and periocular mesenchyme. Proc Natl Acad Sci U S A.

[65] Matt N., Dupé V., Garnier J.-M., Dennefeld C., Chambon P., Mark M., Ghyselinck N.B. (2005). Retinoic acid-dependent eye morphogenesis is orchestrated by neural crest cells. Dev Camb Engl.

[66] Matt N., Ghyselinck N.B., Pellerin I., Dupé V. (2008). Impairing retinoic acid signalling in the neural crest cells is sufficient to alter entire eye morphogenesis. Dev Biol.

[67] Antonica F., Kasprzyk D.F., Opitz R., Iacovino M., Liao X.-H., Dumitrescu A.M., Refetoff S., Peremans K., Manto M., Kyba M. (2012). Generation of functional thyroid from embryonic stem cells. Nature.

[68] Takebe T., Sekine K., Enomura M., Koike H., Kimura M., Ogaeri T., Zhang R.-R., Ueno Y., Zheng Y.-W., Koike N. (2013). Vascularized and functional human liver from an iPSC-derived organ bud transplant. Nature.

[69] Marlow F., Zwartkruis F., Malicki J., Neuhauss S.C., Abbas L., Weaver M., Driever W., Solnica-Krezel L. (1998). Functional interactions of genes mediating convergent extension, knypek and trilobite, during the partitioning of the eye primordium in zebrafish. Dev Biol.

